# Risk assessment tools to predict location of discharge and need for supportive services for medical patients after discharge from hospital: a systematic review protocol

**DOI:** 10.1186/s13643-016-0401-7

**Published:** 2017-01-17

**Authors:** Daniel M. Kobewka, Daniel McIsaac, Michaël Chassé, Kednapa Thavorn, Sunita Mulpuru, Luke T. Lavallée, Shane English, Justin Presseau, Alan J. Forster

**Affiliations:** 1 Department of Medicine—The Ottawa Hospital, University of Ottawa, Ottawa, Ontario Canada; 2 Department of Anesthesiology and Critical Care, Laval University, Quebec, Canada; 3 Clinical Epidemiology Program, Ottawa Hospital Research Institute, Ottawa, Canada; 4 School of Epidemiology, Public Health and Preventive Medicine, University of Ottawa, Ottawa, Ontario Canada; 5 Performance Measurement, The Ottawa Hospital, Ottawa, Ontario Canada; 6 Institute for Clinical Evaluative Sciences, Toronto, Ontario Canada; 7 Department of Medicine, Division of General Internal Medicine, Ottawa Hospital, Civic Campus, 1053 Carling Avenue, Ottawa, Canada

## Abstract

**Background:**

Patients who are discharged from hospital after an acute medical illness often have impaired function that prevents them from returning to their previous place of residence. Assessing each patient’s post-discharge needs takes time and resources but is important in order to reduce unplanned readmissions and adverse events post-discharge.

**Methods/design:**

We will conduct a systematic review to synthesize the evidence on prognostic models and their reported accuracy in predicting the location of discharge after a medical admission to an acute care hospital. We will perform searches in MEDLINE, EMBASE, CINAHL, and COCHRANE databases. Pre-defined study, population, and model characteristics will be reported. We will write a narrative summary of included studies. Methodological quality of the studies will be assessed using the QUIPS tool, and the quality of evidence will be evaluated using the GRADE tool.

**Discussion:**

Early and accurate assessment of patient needs for supportive services after discharge has the potential to improve patient outcomes and health system efficiency. This systematic review will identify factors that can accurately predict location of discharge using existing tools and identify priority knowledge gaps to inform future research.

**Systematic review registration:**

PROSPERO CRD42016037144

**Electronic supplementary material:**

The online version of this article (doi:10.1186/s13643-016-0401-7) contains supplementary material, which is available to authorized users.

## Background

Patients discharged from hospital often have impaired ability to perform instrumental activities of daily living (IADLs—e.g., meal preparation, managing finances, or house work) and activities of daily living (ADLs—e.g., dressing, bathing, and toileting) [1, 2]. While some patients eventually recover to their pre-hospital level of function, many never do [3]. This is especially true for elderly patients with multiple comorbidities, who often require community-based supportive services, or transition to a long-term care facility to meet their care needs [4]. In fact, 30–50% of elderly patients do not return to their functional baseline at 3 months after discharge [3, 5, 6].

Matching a patient’s need for assistance to appropriate support is important as it can minimize the risk of unplanned readmissions and adverse events post-discharge [7, 8]. However, assessing functional status requires resources. Furthermore, it often takes considerable time for the appropriate community service or facility to be arranged or become available for each patient. Some patients in hospital do not even require acute care services at the time of admission but only come to hospital because of gradual decline in their function due to chronic disease or advanced age. In Canada, 14% of all acute care hospital days are used by patients who no longer require hospital care but cannot return to their previous place of residence, due to a temporary or permanent change in their functional status [9].

Early prediction of a patient’s required discharge support services could improve patient care and efficiency in the health system. There are a number of tools that can be used to predict the location of discharge or the level of supportive services required after discharge, but it is not clear which tool to select in a particular circumstance or how these tools compare [3, 5, 10, 11]. A previous systematic review found that advanced age, lower functional status at admission, cognitive impairment, length of stay, and depression were predictors of functional decline after discharge from hospital [12]. The prognostic models identified in the review were of limited clinical utility because of poor to fair predictive accuracy and unknown reliability. Several models predicting the risk of institutional discharge have been implemented for specific diagnoses such as stroke and femur fracture, but it is unknown if these models can be generalized to a heterogeneous group of medical patients [13, 14]. The purpose of this systematic review is to identify and describe existing prognostic models to determine the degree of supportive services required after discharge from hospitals and summarize their reported accuracy.

### Objectives

The primary objective of this systematic review is to identify, describe, and synthesize knowledge on prognostic models that predict the degree of supportive services required after hospitalization, among patients admitted non-electively to a hospital medical service. The secondary objective is to identify variables that were used to predict the level of support required after discharge.

## Methods/design

The systematic review protocol was guided by the Preferred Reporting Items for Systematic Reviews and Meta-analyses Protocol (PRISMA-P) [15, 16]. Our approach will include, in addition to our research goals and objectives, a thorough process for study identification, selection and data abstraction, a methodological quality assessment of included studies, data-analysis, and interpretation. Quality of the evidence will be evaluated using the Grading of Recommendations Assessment, Development and Evaluation (GRADE) methodology.

### Study registration

This systematic review protocol is registered at PROSPERO (Protocol No. CRD42016037144).

### Data sources and literature search

A comprehensive and systematic search strategy will be designed with the assistance of an information specialist. We will use medical subject headings (MeSH) terms and free text terms representing the included study types, population, and outcomes, to be sensitive and inclusive. Our search strategy formatted for MEDLINE can be found in Additional file 1. We will search computerized databases, including MEDLINE, CINAHL, EMBASE, and COCHRANE databases from inception to present. There will be no limitation of the search strategy based on language. Reference lists of published systematic reviews, and eligible studies, will be searched for additional references.

### Study screening and inclusion

#### Study selection:

The title and abstract from all references will be screened independently by two reviewers using the pre-defined inclusion and exclusion criteria. If an abstract is not available, full-text articles will be obtained unless the title is clearly irrelevant. Full-text copies of relevant reports will then be obtained and reviewed independently by two reviewers for final inclusion decision. Two independent reviewers will abstract data from included studies using a standardized data abstraction form. Disagreements will be resolved by consensus and by consultation with a third independent reviewer when needed. Distiller SR® (Ottawa, Canada) will be used to manage screening and data extraction processes. All screening and data extraction forms will be piloted to ensure accuracy and agreement between reviewers.

### Inclusion criteria

#### Study type

We will only include studies that use a prognostic model to assess predictive variables or risk factors and their effects on the outcome of discharge location. We will include both prospective and retrospective studies.

#### Population

We will include studies that examine adult patients (≥18 years of age) admitted non-electively to a medical inpatient service. We will include studies that included patients admitted to a general medicine ward, sub-specialty medicine ward or patients admitted with a specific diagnosis that could be cared for on a general medical ward.

#### Outcomes and setting

The outcome of interest for this review is discharge location. This includes, but is not limited to, home, home with supportive services, rehabilitation, nursing home, hospice, or death. The setting of interest is acute care hospitals.

### Exclusion criteria

Patients admitted to rehabilitation hospitals or to geriatric rehabilitation units will be excluded. Studies that include mixed populations whereby less than 50% of study participants are medicine patients or where medicine specific data cannot be extracted will be excluded. Patients admitted for surgery, maternal, or psychiatric care or diagnoses that would not be cared for on a medical ward will be excluded.

### Data extraction

A standard data extraction form will be prepared a priori and piloted prior to duplicate extraction by two independent reviewers. Data elements, chosen based on clinical knowledge and the Checklist for critical Appraisal and data extraction for systematic Reviews of prediction Modeling Studies (CHARMS) will include [17] the following:

- *Study characteristics, design, and methods*: title, authors, journal/source, year and language of publication, country of conduct, study period, study eligibility criteria, total number of patients in derivation and validation cohorts, data sources (primary data collection or routinely collected administrative data), role of person performing data collection (clinical staff or research assistant), and hospital characteristics (e.g., private vs. public)
- *Population characteristics*: age, sex distribution, admitting diagnosis/diagnoses, prevalence of multimorbidity, prevalence of functional limitation, prevalence of cognitive dysfunction, prevalence of frailty, length of stay, admitting service, hospital, time-frame that predictive variables were collected, and prevalence of institutional living environment prior to hospitalization.
- *Study-specific outcomes*: description of discharge location categories reported or the types of support provided to patients on discharge, number of patients in each discharge location category, and amount of missing data.
- *Model characteristics*: model validation (external/internal/none), variables tested for model building, variables included in the final model, effect size and significance level for each prognostic variable, prevalence/distribution of prognostic variables, measures of discrimination, calibration, overall observed vs. expected performance, reclassification indices, and goodness of fit

### Analysis plan

We will describe study characteristics, patient characteristics, risk of bias assessment, and methodological quality of studies and summarize the reported outcomes of the included studies in a narrative format.

#### Risk of bias assessment

We will use the Quality in Prognosis Studies (QUIPS) tool to assess the methodological quality of each included study [18]. Two independent reviewers will apply the tool to each study. Disagreement about ratings will be resolved by consensus or by consultation with a third independent reviewer when needed. Results from the risk of bias assessment will be presented in table format with color-coding for easy visualization.

#### Primary analysis

The primary analysis will be a narrative summary of models that predict discharge disposition for acute medical patients. We will discuss the variables included in the final models, how the variables were coded, the predictive accuracy of the model, and whether the model was internally or externally validated. We will write a narrative summary of the evidence for each prognostic factor. We will also create a table summarizing the strength of evidence for each prognostic factor. If multiple studies are found that use the same model, then we will meta-analyze the performance of the models. If we perform meta-analysis, we will state that the analysis was a post hoc decision.

#### Sensitivity analyses

A sensitivity analysis will be performed on studies with higher methodological quality that include a heterogeneous group of medical patients, are validated (either internal or external), and receive a rating other than “high bias” in at least one category of the QUIPS tool. This analysis will be a narrative summary that covers the same elements as the primary analysis.

#### Quality of evidence

We will use the GRADE tool that has been adapted for use in narrative systematic reviews of prognostic studies to assess meta-biases and the overall strength of the body of evidence. [19]. We will use the adapted version of GRADE to rate the quality of evidence for each prognostic factor as high, moderate, low, or very low. The strength of evidence for each prognostic factor will be presented in table format with a separate table for each outcome including, discharge to long-term care facility, discharge home, and discharge home with supportive services.

## Discussion

We anticipate that the results of our review will have at least two important impacts on the care of acutely ill medical patients. First, through systematic identification, risk of bias assessment, and subsequent knowledge synthesis, our study will inform current patient care. Predicting care needs at discharge has the potential to improve patient experience and outcomes, as well as health system efficiency. We will disseminate our findings though traditional means, such as peer-reviewed publication, as well as through social media platforms. Furthermore, results will be shared with key stakeholders, such as the Council of Academic Hospitals of Ontario, Health Quality Ontario, and the Canadian Frailty Network to promote uptake across the healthcare system.

Secondly, our review will identify high priority knowledge gaps regarding prediction of discharge disposition and need for support services that will inform future research. Identification of under-represented populations and under-studied outcomes, as well as methodological gaps, will help to frame novel research questions and methods to improve clinicians’ abilities to map scarce resources to patient needs and to ultimately optimize care. Identifying variables with consistent prognostic value and promising underused variables will aid researchers and hospital management teams to develop more accurate models to support discharge decisions. Lastly, our work may help to determine if discharge location can be accurately predicted from routinely collected administrative data or if primary data collection is needed to improve model accuracy.

## Additional file

Additional file 1: Search strategy used for MEDLINE and adapted for other databases. (DOCX 66 kb)
