# Supplementary material for: Risk assessment tools to predict location of discharge and need for supportive services for medical patients after discharge from hospital: a systematic review protocol
Source: Syst Rev. 2017 Jan 17;6:8. doi: 10.1186/s13643-016-0401-7 (PMC5240308; doi:10.1186/s13643-016-0401-7)
Supplement: Additional file 1: — Search strategy used for MEDLINE and adapted for other databases. (DOCX 66 kb) [file 13643_2016_401_MOESM1_ESM.docx]

**Additional file 1**: Search strategy used for MEDLINE and adapted for other databases.

**Database: Ovid MEDLINE(R) In-Process & Other Non-Indexed Citations and Ovid MEDLINE(R) <1946 to Present>**

**Search Strategy:**

--------------------------------------------------------------------------------

1     Hospitalization/ (79436)

2     (hospitali?ed or hospitali?ation).tw,kw. (159878)

3     hospital admission$.tw. (26705)

4     or/1-3 (222986)

5     Patient Discharge/ (21050)

6     discharge.tw,kw. (132064)

7     5 or 6 (139700)

8     aftercare/ or (aftercare or posthospital care).tw,kw. (8383)

9     Subacute Care/ (762)

10     ((subacute or postacute or post acute) adj2 (care or service$)).tw,kw. (1211)

11     exp Nursing Homes/ or (nursing home or nursing facilit$).tw,kw. (40178)

12     retirement home$.tw,kw. (200)

13     ((intermediate or residential or institutional) adj1 care).tw,kw. (5046)

14     Progressive Patient Care/ or progressive patient care.tw,kw. (1213)

15     residential facilities/ or assisted living facilities/ or homes for the aged/ (17381)

16     ((residential or assisted living) adj2 facilit$).tw,kw. (1925)

17     "old age home".tw,kw. (116)

18     Nurses, Community Health/ (184)

19     (nurs$ adj2 visit$).tw,kw. (2436)

20     Hospices/ or hospice$.tw,kw. (10696)

21     Rehabilitation Centers/ or (rehabilitation adj1 (facilit$ or centre$ or center$)).tw,kw. (12059)

22     Patient Transfer/ or patient transfer$.tw,kw. (6990)

23     or/8-22 (92814)

24     Risk factors/ or risk$.tw,kw. (1759139)

25     Risk Assessment/ (196496)

26     "predictive value of tests"/ or roc curve/ or (predict$ or ROC).tw,kw. (1177678)

27     Prognosis/ (395993)

28     case mix adjustment.tw,kw. (354)

29     models, statistical/ or multivariate analysis/ or "analysis of variance"/ (370033)

30     (logistic adj2 (regression or model$)).tw,kw. (171597)

31     (multivariate adj2 (model$ or analysis or regression)).tw,kw. (166687)

32     (prognostic adj5 (identif$ or indicator$ or criteria or scor$ or factor$ or model$)).tw,kw. (93120)

33     (score or scoring system or index$ or rule).ti. (85306)

34     or/24-33 (3336453)

35     4 and 7 and 23 and 34 (893)

36     (child/ or infant/) not adult/ (1145976)

**37     35 not 36 (864)**
